# Berberine Reduces the Metastasis of Chondrosarcoma by Modulating the ****α****v****β****3 Integrin and the PKC****δ****, c-Src, and AP-1 Signaling Pathways

**DOI:** 10.1155/2013/423164

**Published:** 2013-08-22

**Authors:** Chi-Ming Wu, Te-Mao Li, Tzu-Wei Tan, Yi-Chin Fong, Chih-Hsin Tang

**Affiliations:** ^1^School of Chinese Medicine, China Medical University, Taichung 404, Taiwan; ^2^Graduate Institute of Basic Medical Science, China Medical University, No. 91, Hsueh-Shih Road, Taichung 404, Taiwan; ^3^Department of Pharmacology, School of Medicine, China Medical University, Taichung 404, Taiwan; ^4^Department of Orthopaedics, China Medical University Hospital, Taichung 404, Taiwan; ^5^Department of Biotechnology, College of Health Science, Asia University, Taichung 413, Taiwan

## Abstract

Chondrosarcoma is a primary malignant bone cancer, with a potent capacity to invade locally and cause distant metastasis, especially to the lungs. Patients diagnosed with chondrosarcoma have poor prognosis. Berberine, an active component of the Ranunculaceae and Papaveraceae families of plant, has been proven to induce tumor apoptosis and to prevent the metastasis of cancer cells. However, the effects of berberine in human chondrosarcoma are largely unknown. In this study, we found that berberine did not induce cell apoptosis in human primary chondrocytes and chondrosarcoma cells. However, at noncytotoxic concentrations, berberine reduced the migration and invasion of chondrosarcoma cancer cells. Integrins are the major adhesive molecules in mammalian cells and have been associated with the metastasis of cancer cells. We also found that incubation of chondrosarcoma cells with berberine reduced mRNA transcription for, and cell surface expression of, the **α**v**β**3 integrin, with additional inhibitory effects on PKC**δ**, c-Src, and NF-**κ**B activation. Thus, berberine may be a novel antimetastasis agent for the treatment of metastatic chondrosarcoma.

## 1. Introduction

Chondrosarcomas are a heterogeneous group of neoplasms that are characterized by the production of cartilage matrix by tumor cells. They are uncommon, malignant, and lethal primary bone tumors that may occur at any age between 10 and 80 years. Approximately two-thirds of the affected patients are males [1], and the tumor usually appears on the scapula, sternum, ribs, or pelvis [2]. Clinically, surgical resection remains the primary mode of therapy for chondrosarcoma. There is a high incidence of fatality associated with this mesenchymal malignancy due to the absence of an effective adjuvant therapy, and therefore, it is important to explore novel remedies [3].

Tumor invasion and metastasis are the main biological characteristics of cancer cells [4]. Mortality in cancer patients principally results from the metastatic spread of cancer cells to distant organs. Tumor metastasis is a highly complex, multistep process, which includes changes in cell-cell adhesion properties [4]. Because integrins expressed on the surface of a cell determine whether the cell can adhere to and survive in a particular microenvironment, the matching of integrins and ligands plays a key role in metastasis [5]. Integrins are a family of transmembrane glycoprotein adhesion receptors that play central roles in the biology of metazoans by controlling cell adhesion, migration, differentiation, and apoptosis. Integrins form heterodimers of *α*- and *β*-subunits [6]. There are at least 19 *α*-subunits and 8 *β*-subunits that can associate to form 25 unique integrin heterodimers [7, 8]. Integrins play an important role in many extracellular matrix (ECM) proteins such as collagens, fibronectin, laminin, osteopontin, and vitronectin [9]. In addition, they have been implicated in the metastasis of chondrosarcomas and lung, breast, bladder, and colon cancers [10–13]. The *α*v*β*3 integrin, in particular, has been reported in chondrosarcoma progression, with effects on angiogenesis, survival, and invasion [14, 15]. *In vitro* studies have also found that the *α*v*β*3 integrin facilitated chondrosarcoma migration and invasion through several ECM substrates and transendothelial migration [16].

Berberine, an active component of the Ranunculaceae and Papaveraceae families of plant, is part of the well-studied group of naturally occurring isoquinoline alkaloids. It has been suggested that the beneficial properties of berberine may also have an effect on other diseases such as diabetes, hypertension, arrhythmia, and gastrointestinal diseases [17]. A recent study has shown its potential chemotherapeutic efficacy against cancers [18]. In addition, berberine has been reported to reduce the metastasis of human gastric cancer, prostate cancer, and breast cancer [19–21]. However, the effects of berberine in the metastasis of human chondrosarcoma cells are largely unknown. Here, we report that berberine inhibits the migration and invasion of human chondrosarcoma cells. In addition, the downregulation of the *α*v*β*3 integrin through protein kinase C (PKC)*δ*, c-Src, and AP-1 is involved in berberine-reduced cell motility. Therefore, our data provide evidence that berberine may be an antimetastatic agent for the treatment of metastasic chondrosarcoma.

## 2. Experimental Section

### 2.1. Materials

Protein A/G beads, rabbit polyclonal antibodies specific for p-c-Jun, and c-Jun were purchased from Santa Cruz Biotechnology (Santa Cruz, CA, USA). The pSV-*β*-galactosidase vector and luciferase assay kit were purchased from Promega (Madison, MA). All other chemicals were purchased from Sigma-Aldrich (St. Louis, MO, USA).

### 2.2. Cell Culture

The human chondrosarcoma cell line JJ012 was kindly provided by the laboratory of Dr. Sean P. Scully (University of Miami School of Medicine, Miami, FL, USA) [22]. Cells were cultured in Dulbecco's Modified Eagle's Medium (DMEM)/*α*-MEM supplemented with 10% fetal bovine serum (FBS). The human chondrosarcoma cell line SW1353 was obtained from the American Type Culture Collection. These cells were cultured in DMEM supplemented with 10% FBS. All cells were maintained at 37°C in a humidified atmosphere of 5% CO_2_.

To establish primary cultures, chondrocytes were isolated from articular cartilage, as previously described [23]. The cells were grown in plastic cell culture dishes in 95% air-5% CO_2_, in DMEM supplied with 20 mM HEPES, 10% heat-inactivated FBS, 2 mM-glutamine, 100 U/mL penicillin, and 100 *μ*g/mL streptomycin.

### 2.3. MTT Assay

Cell viability was determined with a 3-(4,5-dimethylthiazol-2-yl)-2,5-diphenyltetrazolium bromide (MTT) assay. After being treated with berberine for 24 or 48 h, the cultures were washed with phosphate-buffered saline (PBS). Then, MTT (0.5 mg/mL) was added to each well, and the mixture was incubated at 37°C for 2 h. To dissolve formazan crystals, the culture medium was replaced with an equal volume of DMSO. After the mixture was shaken at room temperature for 10 min, the absorbance of each well was determined at 550 nm by using a microplate reader (Bio-Tek, Winooski, VT, USA).

### 2.4. TUNEL Assay

Cell apoptosis was examined through a terminal deoxynucleotidyl transferase-mediated deoxyuridine triphosphate nick-end labeling (TUNEL) assay performed using the BD ApoAlert DNA Fragmentation Assay Kit. Cells were incubated with berberine for 24 h, then trypsinized, fixed with 4% paraformaldehyde, and permeabilized with 0.1% Triton-X-100 in 0.1% sodium citrate. After being washed, the cells were incubated with the reaction mixture for 60 min at 37°C. The stained cells were then analyzed with a flow cytometer.

### 2.5. Caspase 3 Activity Assay

This assay is based on the ability of an active enzyme to cleave a chromophore from the enzyme substrate Ac-DEVD-pNA. Cell lysates were prepared and incubated with anti-caspase 3. Immunocomplexes were incubated with the peptide substrate in assay buffer (100 mM NaCl, 50 mM 4-(2-hydroxyethyl)-1-piperazine-ethanesulphonic acid [HEPES], 10 mM dithiothreitol, 1 mM EDTA, 10% glycerol, and 0.1% 3-[(3-cholamidopropyl) dimethylammonio]-1-propanesulfonate (CHAPS), pH 7.4) for 2 h at 37°C. The release of *p*-nitroaniline was monitored at 405 nm. The results are the percent change in activity compared to the untreated control.

### 2.6. Migration and Invasion Assay

The migration assay was performed using Transwell inserts (Costar, NY; 8 mm pore size) in 24-well dishes. For the invasion assay, filters were precoated with 30 *μ*L Matrigel basement membrane matrix (BD Biosciences, Bedford, MA) for 30 min. The following procedures were the same for both migration and invasion assays. After treatment with berberine (0, 1, 3, 10, and 30 *μ*M) for 24 h, cells were harvested and seeded to Transwell at 1 × 10^4^ cells/well in serum-free medium, and then incubated for 24 h at 37°C in 5% CO_2_. Cells were then fixed in 3.7% formaldehyde for 5 min and stained with 0.05% crystal violet in PBS for 15 min. Cells on the upper side of the filters were removed with cotton-tipped swabs, and the filters were washed with PBS. Cells on the underside of the filters were examined and counted under a microscope. Each experiment was performed in triplicate and repeated at least three times. 

### 2.7. Wound-Healing Migration Assay

For the wound-healing migration assay, cells were seeded on 12-well plates at a density of 1 × 10^5^ cells/well in culture medium. Twenty-four hours after seeding, the confluent monolayer of culture was scratched with a fine pipette tip, and migration was visualized by microscope. The rate of wound closure was observed at the indicated time.

### 2.8. Flow Cytometric Analysis

Human chondrosarcoma cells were grown in 6-well dishes, and then washed with PBS and detached using trypsin at 37°C. Cells were fixed for 10 min in PBS containing 3.7% paraformaldehyde, rinsed in PBS and incubated with mouse anti-human *α*v*β*3 integrin (1 : 100) (BD Biosciences, CA, USA) for 1 h at 4°C. Cells were then washed in PBS, and incubated with fluorescein isothiocyanate-conjugated goat anti-mouse secondary IgG (1 : 100; Leinco Technologies, St Louis, MO) for 45 min at 4°C. After a final rinse, cells were analyzed using a FACSCalibur flow cytometer and CellQuest software (BD Biosciences, CA).

### 2.9. Western Blot Analysis

Cellular lysates were prepared, and proteins were resolved by SDS-PAGE [24, 25]. Proteins were then transferred to Immobilon polyvinylidene fluoride membranes. The blots were blocked with 4% bovine serum albumin for 1 h at room temperature and probed with rabbit anti-human antibodies against p-c-Jun or c-Jun (1 : 1000) for 1 h at room temperature (Santa Cruz, CA). After three washes, the blots were incubated with peroxidase-conjugated donkey anti-rabbit secondary antibody (1 : 1000) for 1 h at room temperature. The blots were visualized with enhanced chemiluminescence by using X-OMAT LS film (Eastman Kodak, Rochester, NY). 

### 2.10. Kinase Activity Assay

PKC*δ* and c-Src activities were assessed with a PKC kinase activity assay kit (Assay Designs, Inc., Ann Arbor, MI) and a c-Src kinase activity assay kit (Abnova, Corp., Taipei, Taiwan). The kinase activity kits are based on a solid-phase ELISA that uses a specific synthetic peptide as substrate for PKC*δ* or c-Src, and a polyclonal antibody that recognizes the phosphorylated form of the substrate.

### 2.11. Quantitative Real-Time PCR

Total RNA was extracted from chondrosarcoma cells by using a TRIzol kit (MDBio, Taipei, Taiwan). Reverse transcription was performed using 1 *μ*g of total RNA and an oligo(dT) primer [26]. Quantitative real-time PCR (qPCR) was carried out using a TaqMan One-step PCR Master Mix (Applied Biosystems, CA, USA). Total cDNA (100 ng) was added to each 25 *μ*L reaction mixture with sequence-specific primers and TaqMan probes. All target gene primers and probes were purchased commercially, including those for GAPDH as an internal control (Applied Biosystems). qPCR was carried out in triplicate with a StepOnePlus (Applied Biosystems) sequence detection system. The cycling conditions were 10 min at 95°C, followed by 40 cycles of 95°C for 15 s, and 60°C for 60 s. To calculate the cycle number at which the transcript was detected (*C*_*T*_), the threshold was set above the nontemplate control background and within the linear phase of target gene amplification.

### 2.12. Reporter Gene Assay

The chondrosarcoma cells were transfected with AP-1 reporter plasmid by using Lipofectamine 2000 according to the manufacturer's recommendations. Twenty-four hours after transfection, the cells were treated with inhibitors for 30 min. Next, berberine or vehicle was added for 24 h. Cell extracts were then prepared, and luciferase and *β*-galactosidase activities were measured.

### 2.13. Statistical Analysis

Data are presented as mean ± standard error of the mean (SEM). Statistical analysis of the two samples was performed using the Student's *t*-test. Statistical comparisons of more than two groups were performed using one-way analysis of variance with Bonferroni's post hoc test. In all cases, *P* < 0.05 was considered significant.

## 3. Results

### 3.1. Berberine Did Not Induce Cell Death in Primary Chondrocytes and Human Chondrosarcoma Cells

**Figure 1:**
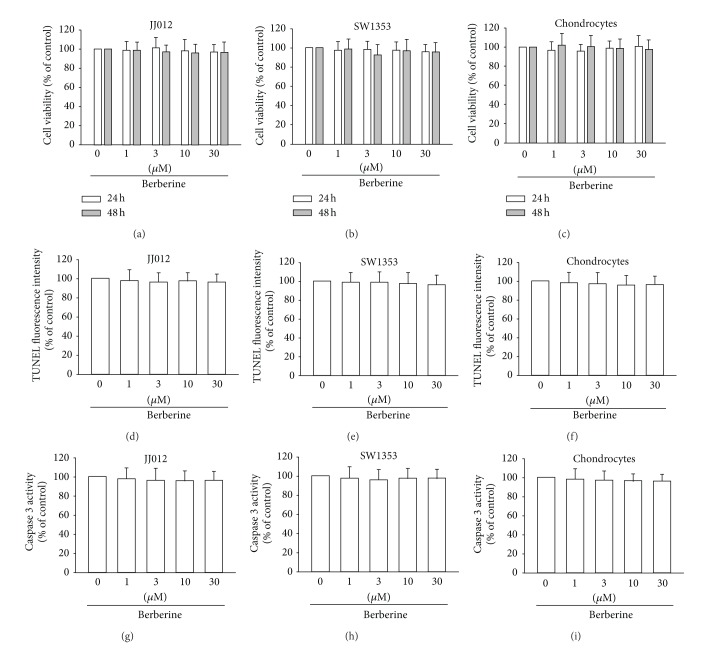
Berberine did not induce cell apoptosis in human chondrocytes and chondrosarcoma cells. ((a)–(c)) Cells were incubated with various concentrations of berberine for 24 or 48 h, and cell viability was examined by MTT assay. ((d)–(f)) Cells were incubated with berberine for 24 h; TUNEL-positive cells were examined by flow cytometry. ((g)–(i)) Cells were incubated with berberine for 24 h, and caspase 3 activity was examined using caspase 3 ELISA kit. Results are expressed as the mean ± S.E.M.*, *P* < 0.05 compared with control.

It has been reported that berberine increases death in human cancer cells [18]. We therefore investigated whether berberine induced cell death in human chondrosarcoma cells. The cytotoxic effect of berberine in chondrosarcoma cells was examined by MTT assay. Stimulation of chondrosarcoma cells (JJ012 and SW1353) for 24 or 48 h did not affect cell viability (Figures 1(a) and 1(b)). Furthermore, berberine also did not affect the cell viability of normal primary chondrocytes (Figure 1(c)). Next, we examined whether berberine induced cell apoptosis in human chondrosarcoma cells by TUNEL staining and caspase 3 activity assays. However, incubation of cells with berberine did not enhance TUNEL expression (Figures 1(d)–1(f)). Berberine also did not affect caspase 3 activity in normal chondrocyte or chondrosarcoma cell lines (Figures 1(g)–1(i)). These data indicate that berberine did not induce cell death in human primary chondrocytes and chondrosarcoma cells. Therefore, we used this berberine concentration range for all subsequent experiments

### 3.2. Berberine Reduces Cell Migration, Wound-Healing Migration, and Cell Invasion in Human Chondrosarcoma Cells

**Figure 2:**
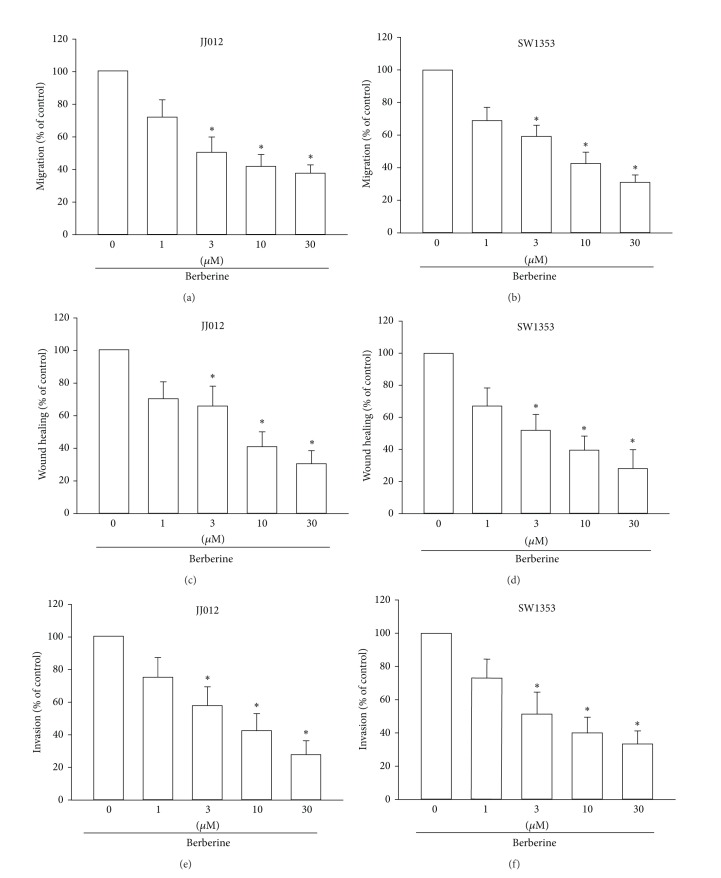
Berberine inhibits migration and invasion of human chondrosarcoma cells. ((a)–(f)) Cells were incubated with various concentrations of berberine for 24 h; cell migration and invasion were examined through Transwell, wound healing, and invasion assays. Results are expressed as the mean ± S.E.M.*, *P* < 0.05 compared with control.

The role of berberine in reducing the metastasis of human cancers has been previously documented [19–21]. Therefore, we next checked whether berberine inhibits cell motility in chondrosarcoma cancer cells. The results from the Transwell migration assay showed that incubation of chondrosarcoma cells with berberine (1–30 *μ*M) dramatically decreased migration in both chondrosarcoma cell lines (Figures 2(a) and 2(b)). The wound-scratching assay also revealed that berberine reduced wound-healing activity in chondrosarcoma cells (Figures 2(c) and 2(d)). In addition, incubation of cells with berberine thwarted the ability of chondrosarcoma cells to invade through a Matrigel basement membrane matrix (Figures 2(e) and 2(f)). These results suggest that berberine suppresses cell migration and invasion in human chondrosarcoma cells.

### 3.3. Berberine Reduces Cell Motility in Chondrosarcoma Cells by Inhibiting the Expression of the *α*v*β*3 Integrin

**Figure 3:**
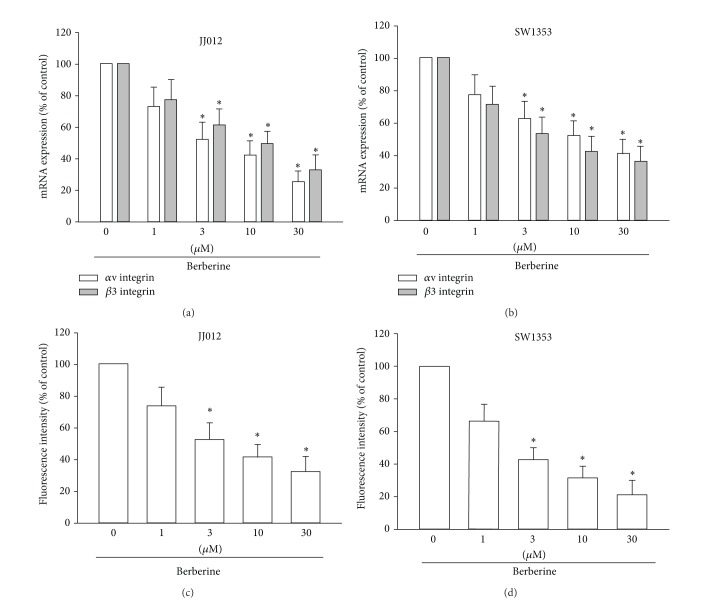
Berberine inhibits expression of the *α*v*β*3 integrin in chondrosarcoma cells. ((a)–(d)) Cells were incubated with various concentrations of berberine for 24 h; mRNA and cell surface expression of *α*v*β*3 integrin were examined by qPCR and flow cytometry. Results are expressed as the mean ± S.E.M.*, *P* < 0.05 compared with control.

Upregulation of the *α*v*β*3 integrin has been known to increase the metastasis of human chondrosarcomas [27]. We therefore hypothesized that the *α*v*β*3 integrin may be involved in berberine-mediated inhibition of migration in human chondrosarcoma cells. We found that incubation of chondrosarcoma cells with berberine reduced the mRNA expression of *α*v and *β*3 integrin in a concentration-dependent manner (Figures 3(a) and 3(b)). Similarly, stimulation of cells with berberine inhibited the cell surface expression of the *α*v*β*3 integrin (Figures 3(c) and 3(d)). These data suggest that berberine reduces the metastasis of chondrosarcoma cells by inhibiting the expression of *α*v*β*3 integrin.

### 3.4. Berberine Reduces the Activity of the PKC*δ* and c-Src Signaling Pathways

**Figure 4:**
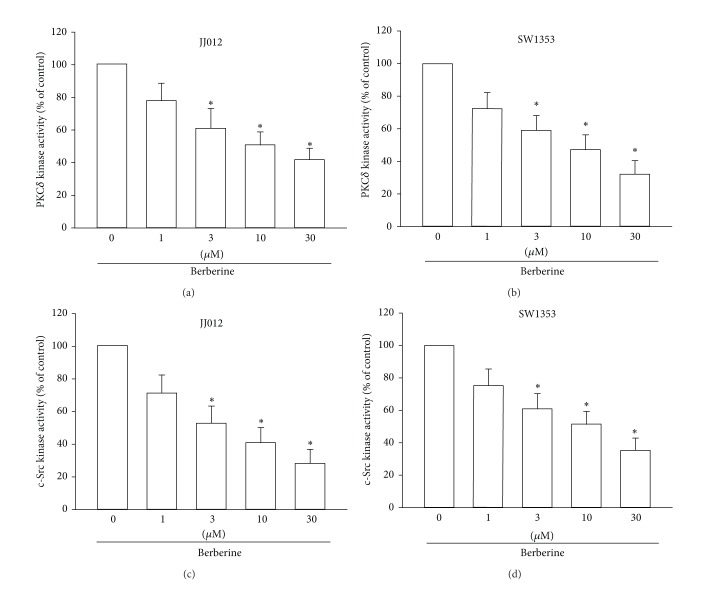
PKC*δ* and c-Src signaling pathways are involved in the berberine response of human chondrosarcoma cells. ((a) and (b)) Cells were incubated with various concentrations of berberine for 24 h; PKC*δ* kinase activity was examined by use of the PKC*δ* kinase activity kit. ((c) and (d)) Cells were incubated with various concentrations of berberine for 24 h; c-Src kinase activity was examined by use of the c-Src kinase activity kit. Results are expressed as the mean ± S.E.M.*, *P* < 0.05 compared with control.

PKC*δ*-dependent c-Src activation has been reported to mediate the metastasis of human oral cancer cells [28]. After the inhibitory effects of berberine on cell migration and integrin expression were revealed, the effects of berberine on the expression of the PKC*δ* and c-Src pathways were investigated. Stimulation of chondrosarcoma cells with berberine reduced PKC*δ* kinase activity in a concentration-dependent manner (Figures 4(a) and 4(b)). Furthermore, berberine also reduced c-Src kinase activity in chondrosarcoma cells (Figures 4(c) and 4(d)). Therefore, berberine appears to act through a signaling pathway involving PKC*δ* and c-Src to inhibit cell migration in human chondrosarcoma cells.

### 3.5. AP-1 Is Involved in Berberine-Mediated Metastasis in Chondrosarcoma Cells

**Figure 5:**
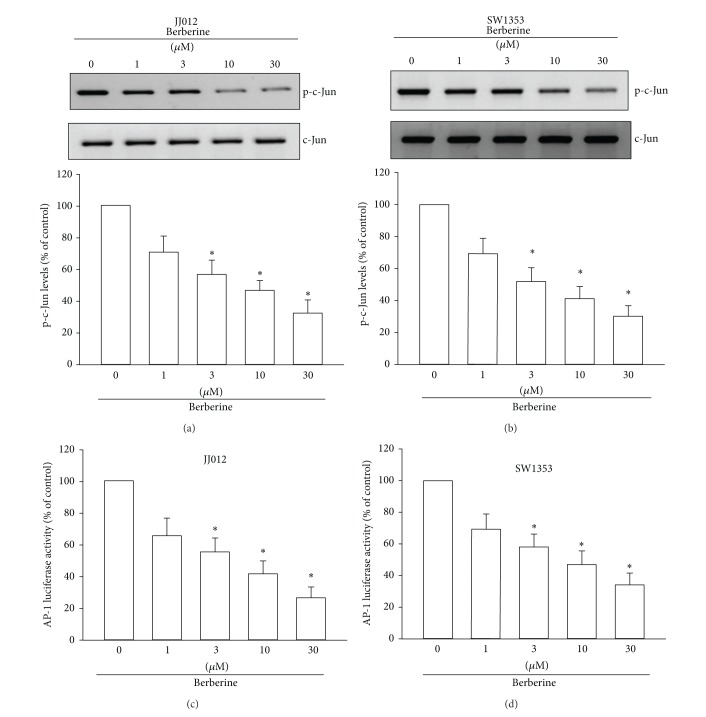
AP-1 mediates the response of human chondrosarcoma cells to berberine. ((a) and (b)) Cells were incubated with various concentrations of berberine for 24 h; p-c-Jun expression was examined by Western blotting. ((c) and (d)) Cells were incubated with various concentrations of berberine for 24 h; AP-1 activity was examined through AP-1 luciferase activity assay. Results are expressed as the mean ± S.E.M.*, *P* < 0.05 compared with control.

AP-1 was found to be involved in the expression of the *α*v*β*3 integrin and the metastasis of chondrosarcoma [16]. So, the role of berberine of AP-1 activation in chondrosarcoma cells was examined. We found that stimulation of cells with berberine inhibited the phosphorylation of p-c-Jun (Figures 5(a) and 5(b)). AP-1 activation was further evaluated by analyzing AP-1 luciferase activity. Cells were transiently transfected with AP-1 luciferase as an indicator of AP-1 activation. We found that berberine reduced AP-1-luciferase activity (Figures 5(c) and 5(d)), implying that AP-1 is involved in berberine-reduced metastasis in chondrosarcoma cells.

## 4. Discussion

Chondrosarcoma is a rare but deadly form of bone cancer. It is the second most common type of bone cancer, accounting for nearly 26% of all bone cancers [29]. The metastatic potential of conventional chondrosarcomas correlates well with the histologic tumor grade because of the relatively indolent growth rates of many low- and moderate-grade chondrosarcomas; approximately 15% of all metastatic disease-related deaths occur more than 5 years after initial diagnosis [30]. Therefore, it is important to develop effective adjuvant therapy to prevent chondrosarcoma metastasis. Berberine has various biological applications for disease, with properties that are antidiabetes, antihypertension, antiarrhythmia, and antigastrointestinal disease [17]. Berberine also has been reported to diminish the metastatic potential of human cancer cells [31]. However, the antimetastasic effects of berberine on chondrosarcoma cells are mostly unknown. In the current study, we found that, at noncytotoxic concentrations (0–30 *μ*M), berberine reduced cell motility in human chondrosarcoma cells. Furthermore, berberine did not increase cell death in primary chondrocytes. We found that the downregulation of the *α*v*β*3 integrin through the PKC*δ*, c-Src, and AP-1 pathways is involved in berberine-reduced cancer migration. In this study, we identified berberine as a potential lead base, with good pharmacological properties, on antimetastasic activity in human chondrosarcoma cells. 

Integrins, which link the extracellular matrix to intracellular signaling molecules, regulate a number of cellular processes, including adhesion, signaling, motility, survival, gene expression, growth, and differentiation [32, 33]. Inhibition of *α*v*β*3 integrin by disintegrin or *α*v*β*3 integrin antibody reduced the metastasis of human cancer cells [34, 35]. Therefore, reducing the expression of *α*v*β*3 integrin is a good target for the treatment of the metastasis of human cancer cells. Here, we reported that berberine reduced the mRNA expression of *α*v and *β*3 integrin. In addition, incubation of chondrosarcoma cells with berberine diminished the cell surface expression of *α*v*β*3 integrin. These results indicate that berberine reduces chondrosarcoma metastasis through the downregulation of *α*v*β*3 integrin expression. 

PKC isoforms have been characterized at the molecular level and have been found to mediate several cellular molecular responses [36]. Of the isoforms, PKC*δ* has been shown to mediate tumor migration and metastasis [28, 37]. In our study, we found that, depending on dosage, berberine reduced PKC*δ* kinase activity in chondrosarcoma cells. These results, therefore, suggest that PKC*δ* is involved in the berberine-mediated cell motility of chondrosarcoma cells. Our results provide evidence that berberine downregulates cell motility and *α*v*β*3 integrin expression in human chondrosarcoma cancer cells by way of the PKC*δ* signaling pathway.

Because PKC*δ*-dependent c-Src activation mediates tumor migration and invasion [28, 37], we examined the potential role of PKC*δ*-dependent c-Src in the signaling pathway of berberine-reduced cell motility. Treatment of chondrosarcoma cells with berberine also eliminated c-Src kinase activity, indicating the involvement of PKC*δ*-dependent c-Src activation in berberine-inhibited expression of the *α*v*β*3 integrin and in the metastasis of human chondrosarcoma cells.

The transcription factors of the Jun and Fos families bind to the AP-1 sequence. These nuclear proteins interact with the AP-1 site as Jun homodimers or Jun-Fos heterodimers that are formed by protein dimerization through their leucine zipper motifs. A variety of growth factors stimulates cancer metastasis via signal-transduction pathways that converge to activate the AP-1 complex of transcription factors [38]. The results of this study show that AP-1 activation contributes to berberine-inhibited migration and metastasis in human chondrosarcoma cells. We found that berberine reduced the phosphorylation of c-Jun. In addition, using transient transfection with AP-1 luciferase as an indicator of AP-1 activity, we found that berberine reduced the activity of AP-1 luciferase. Our data indicate that AP-1 activation might play an important role in cell migration and the metastasis of human chondrosarcoma cells. A variety of growth factor stimulate cancer metastasis via signal-transduction pathways that converge to activate NF-*κ*B complex of transcription factors [39]. NF-*κ*B has been reported to regulate the metastasis of human chondrosarcoma [40]. However, we did not examine the role of NF-*κ*B in berberine-inhibited metastasis of human chondrosarcoma in the current study. Therefore, whether NF-*κ*B mediated berberine-inhibited metastasis needs further examination. 

**Figure 6:**
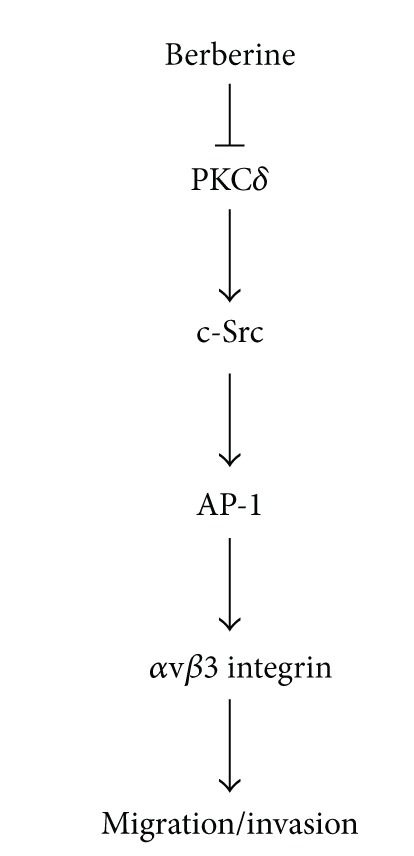
Schematic presentation of the signaling pathways involved in berberine-inhibited metastasis of human chondrosarcoma. Berberine inhibits the migration and invasion of human chondrosarcoma cells by modulating the *α*v*β*3 integrin through PKC*δ*, c-Src, and AP-1 signaling pathway.

It has been recommended that drugs made from natural products play a dominant role in pharmaceutical care. Natural products are important sources of potential agents of cancer chemotherapy and metastasis [41]. The present study showed that berberine inhibits the migration and invasion of human chondrosarcoma cancer cells and that the downregulation of *α*v*β*3 integrin through the PKC*δ*, c-Src, and AP-1 pathways is involved in carrying out berberine-mediated effects (Figure 6). The evidence signals that berberine may show beneficial effects in reducing the metastatic activity of human chondrosarcoma cells.
